# 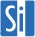
Dynamic information processing states revealed through neurocognitive models of object semantics

**DOI:** 10.1080/23273798.2014.970652

**Published:** 2014-10-13

**Authors:** Alex Clarke

**Affiliations:** ^a^ Department of Psychology, University of Cambridge, Cambridge, UK

**Keywords:** concepts, feature-based, coarse-to-fine, perception

## Abstract

Recognising objects relies on highly dynamic, interactive brain networks to process multiple aspects of object information. To fully understand how different forms of information about objects are represented and processed in the brain requires a neurocognitive account of visual object recognition that combines a detailed cognitive model of semantic knowledge with a neurobiological model of visual object processing. Here we ask how specific cognitive factors are instantiated in our mental processes and how they dynamically evolve over time. We suggest that coarse semantic information, based on generic shared semantic knowledge, is rapidly extracted from visual inputs and is sufficient to drive rapid category decisions. Subsequent recurrent neural activity between the anterior temporal lobe and posterior fusiform supports the formation of object-specific semantic representations – a conjunctive process primarily driven by the perirhinal cortex. These object-specific representations require the integration of shared and distinguishing object properties and support the unique recognition of objects. We conclude that a valuable way of understanding the cognitive activity of the brain is though testing the relationship between specific cognitive measures and dynamic neural activity. This kind of approach allows us to move towards uncovering the information processing states of the brain and how they evolve over time.

Recognising objects is fundamental to acting appropriately in the environment. The rapid extraction of semantic information from visual images is one of the first cognitive operations leading to complex behaviours such as object identification, object use and navigational planning. Extracting semantic information from our visual world necessitates that sensory information is transformed into more abstract, meaningful information. Such transitions from perception to semantics remain unclear in terms of the types of information that are processed, and how this information changes and develops over time. Critical to understanding this complex informational transformation is the use of computational and cognitive models of vision and semantics to reveal what time-varying neural activity reflects in terms of information processing states and mental representations. This is illustrated using three examples using magnetoencephalography (MEG), a neuroimaging technique that records neural responses with millisecond temporal resolution and allows us to track how objects are processed over time. Finally, I chart the time-course of activating semantic knowledge during object recognition focusing on three main issues: how the initial perceptual information processing states give rise to semantic information, what is the nature of this semantic information and how do semantic representations change over time.

## Modelling information processing states in the brain

The observed brain activity at a large-scale population level of the system – whether it comes from functional magnetic resonance imaging (fMRI), MEG, electroencephalography (EEG), positron emission tomography (PET), etc. –can be considered to be a manifestation of how particular aspects of the stimuli are encoded in the brain. In this respect, mental representations reflect the relationship between particular aspects of the stimuli and dynamic activity in the brain – in other words, a *representation* can be viewed as reflecting the current information processing state of the brain, and will be constrained as a function of the regional inputs, environmental constraints and computational properties of the particular brain region.

One approach to uncovering the information processing states of the brain is to have an explicit model of the experimental conditions or task, and to determine to what extent the model can explain the observed data. To illustrate, consider the brain activity patterns within the posterior fusiform gyrus for a set of visual objects. The variability in activation between objects is a vital source of information about the nature of information processing in this region, and elucidating the properties of the experimental conditions that track this variability is key to understanding the cognitive functions of the region. By developing models of the stimulus we can explicitly quantify various aspects of the stimulus, and test if these attributes are reflected in brain activity. Finding a significant relationship between the stimulus attributes and the brain responses can be used to infer what specific forms of information are processed by a particular brain region. Clearly this requires that we have a cognitive or computational model of our stimuli with which to interpret such variability. It is here that multidimensional models of object semantics can be highly informative in uncovering what kind of information processing gives rise to the mental representations of visual objects observed in human neuroimaging. Further, using techniques like MEG we can ask highly detailed questions about when a given brain region processes specific forms of information and how the information content changes over time (see Schyns, Petro, & Smith, 2007 for example). Thus by combining information rich stimulus information with time-varying neural signals we can begin to uncover the dynamic information processing states underlying object recognition.

## The dynamic nature of semantic processing: evidence from MEG and feature-based models of conceptual representations

The primary research question discussed here concerns how different cognitive aspects of object recognition are processed in the brain, and how this changes and evolves over time. One particularly useful approach to obtaining a window into the information processing states underlying meaningful object recognition is provided by formulating detailed, multidimensional cognitive accounts of object semantics which provide a rich source of information that can be tested against neural activity across space and time.

To understand how different properties of objects are processed over time we have dissected our stimuli (objects) into their visual and semantic attributes. While visual image statistics can be extracted from the pictorial images, cognitive accounts of object semantics are needed to extract measures of object meaning. The approach we have taken to representing the semantics of individual objects is provided by models where semantic representations are compositional in nature, being represented in a distributed system of semantic primitives (Cree & McRae, 2003; Farah & McClelland, 1991; Garrard, Lambon Ralph, Hodges, & Patterson, 2001; Humphreys, Lamote, & Lloyd-Jones, 1995; Moss, Tyler, & Taylor, 2007; Rogers & McClelland, 2004; Rogers & Patterson, 2007; Taylor, Devereux, & Tyler, 2011; Tyler & Moss, 2001; Vigliocco, Vinson, Lewis, & Garrett, 2004). The derived semantic features can come from property norming data, where participants are asked to list conceptual properties, or features, of each concept (e.g. *has legs* is a feature of a cow; Devereux, Tyler, Geertzen, & Randall, 2013; McRae, Cree, Seidenberg, & McNorgan, 2005). Semantic features derived from large-scale property norming studies have proven to be a useful way of estimating a concept's semantic content and their internal topological structure – determined by statistical properties calculated across features, such as feature interconnectedness (McRae, de Sa, & Seidenberg, 1997; Moss et al., 2007; Rosch, Mervis, Gray, Johnson, & Boyes-Braem, 1976; Taylor, Devereux, Acres, Randall, & Tyler, 2012; Tyler & Moss, 2001). As such, models based on measures derived from semantic features might be particularly suited to understanding the information processing demands in the brain over time and is outlined in more detail below.

By relating feature-based models of object semantics to MEG signals over time, it is possible to ask whether different forms of information are reflected in neural activity, before establishing *when* different forms of information are processed. I will first show that semantic information, based on semantic features, can be used to successfully model neural activity over time when we recognise objects, and that different kinds of semantic distinctions can be made at relatively early and late latencies. Second, how a specific cognitive model of semantic knowledge – the conceptual structure account (CSA; Moss et al., 2007; Taylor et al., 2011; Tyler & Moss, 2001) –can be used to uncover exactly what forms of semantic information underlie meaningful object processing over time. The third study further shows what kind of neural mechanisms underlie the formation of object-specific semantic information as time elapses.

**Figure 1.**
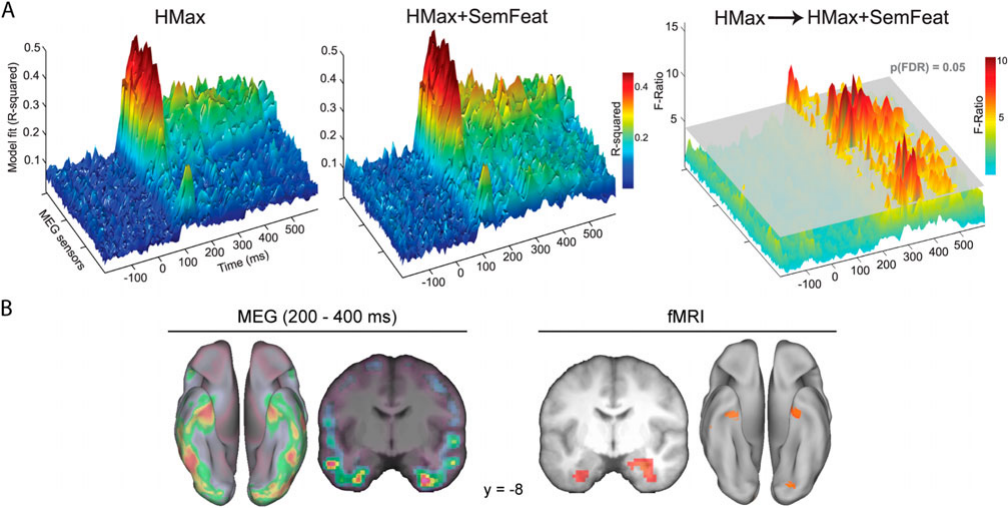
The temporal and spatial distribution of object-specific semantic feature information. (a) Model fit between the HMax model (left) and the combined HMax and semantic feature model (centre) to the MEG data over sensors and time. Right: significant increases in model fit are observed from 190 ms when including semantic feature information in addition to the HMax model. (b) Spatial distribution of semantic feature effects from MEG and fMRI, showing a correspondence in the anterior temporal lobes. MEG data in ‘a’ and ‘b’ reproduced from Clarke et al. (2014), fMRI data in ‘b’ reproduced from Clarke and Tyler (2014).

Clarke, Devereux, Randall, and Tyler (2014) defined visual and semantic parameters for a large set of objects based on a biologically inspired computational model of immediate vision (the HMax model; Riesenhuber & Poggio, 1999; Serre, Wolf, Bileschi, Riesenhuber, & Poggio, 2007) and semantic feature information from a property norming study (McRae et al., 2005). They started with a stimulus model that only included visual parameters capturing the function of V1/V2 cells, and showed that this model could account for MEG signals peaking around 100 ms. Further, adding higher-level visual information (parameters that model posterior inferior temporal cortex) to the model significantly improved the fit between the model and the observed MEG data between 100 and 150 ms. Critically, adding semantic feature information improved model fit from 190 ms – showing that semantic information can capture important aspects of object representations that are not accounted for by computational models of vision alone (Figure 1a). The semantic feature effects were seen to localise to the anterior temporal and posterior ventral temporal lobes, showing similar localisation of semantic feature effects for objects to those observed in the perirhinal cortex as measured by fMRI (Figure 1b; Clarke & Tyler, 2014).

Importantly, Clarke et al. (2014) could also ask at what latencies are sufficient information available to distinguish between objects from different superordinate categories (e.g. between an animal and a tool), and between objects from the same category (e.g. a lion vs. a tiger). They found that semantic feature information could successfully drive between-category distinctions from 110 ms, and within-category distinctions from 150 ms. As classification success is based on the specific predictors in the model, this study shows the validity of using semantic feature information to model time-varying neural representations recorded by MEG, while showing that different aspects of conceptual representations are processed over time – early processing of coarse semantic information and later processing of object-specific semantic information. However, this study does not uncover what kind of semantic information drives this coarse and fine-grained semantic trajectory. To uncover the exact form of information processing requires that we have a cognitive model of conceptual processing that is more detailed than modelling semantic feature content alone.

The statistical regularities between features and feature-types have been an important influence on the development of different cognitive models of conceptual representations. Like many distributed accounts of conceptual knowledge, the CSA (Moss et al., 2007; Taylor et al., 2011; Tyler & Moss, 2001) claims that conceptual representations are composed of distributed and interconnected feature primitives, and that the statistical regularities between features play a vital role during the activation of conceptual knowledge (see Mahon & Caramazza, 2009; Taylor et al., 2011 for reviews and alternative models). The unique contribution of the CSA is in highlighting the importance of the interaction of different feature statistics (Randall, Moss, Rodd, Greer, & Tyler, 2004; Taylor et al., 2012) and instantiating these processes in a neurobiological model of object recognition (Tyler et al., 2013).

Statistical regularities derived from semantic features provide an internal topological structure that influences the ease and speed of activating concept-level representations, which correlate with behavioural performance on a variety of semantic tasks (Cree, McNorgan, & McRae, 2006; Gonnerman, Andersen, Devlin, Kempler, & Seidenberg, 1997; McRae et al., 1997; Randall et al., 2004; Taylor et al., 2012; Taylor, Salamoura, Randall, Moss, & Tyler, 2008). Semantic features can occur in a variable number of concepts, and can be loosely distinguished as being shared by many other concepts (e.g. *has ears*, *has legs* are features shared by many animals) or more distinctive of a particular concept (e.g. *has a hump* for a camel). Concepts with many shared features are, by definition, similar to many other concepts and so require increased conceptual processing to individuate them from their semantic neighbours. Further, processing related to this shared feature information can be informative of the objects category membership, while having more distinctive features results in fewer similar concepts and facilitates the activation of a unique conceptual representation. A second feature-statistic is correlational strength, which captures how often a concept's features tend to co-occur together across concepts. Greater correlation between a concept's features strengthens the links between them, speeding their co-activation and facilitating conceptual processing. Therefore such feature-based statistics could prove key in understanding the exact form of semantic information processed over time. It is also worth noting that these semantic features are not proposed to be literally encoded in neural activity, but rather they provide a model of semantic content and a means by which to estimate statistical regularities in semantic knowledge.

**Figure 2.**
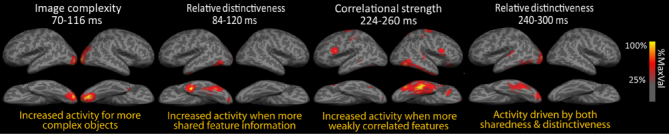
Modulation of object processing by visual and semantic feature-based statistics over time. Data show rapid visual and shared semantic-feature effects before later effects of both shared and distinctive semantic features. Redrawn from Clarke et al. (2013).

Clarke, Taylor, Devereux, Randall, and Tyler (2013) tested for the influence of such feature-based statistics on time-varying neural activity, again using MEG (Figure 2). This approach revealed that semantic feature-statistics rapidly modulated neural activity, and showed that early signals were sensitive to the visual and semantic characteristics of objects. The authors report evidence that a specific type of semantic information is processed following the initial visual effects. Specifically, they showed that MEG signals increased for objects with a greater degree of shared semantic feature information within the first 150 ms. Later, between 200 and 300 ms, neural activity was seen to reflect processing of both shared and distinctive feature information – shown by both increasing MEG signals for objects with more shared feature information and increasing MEG signals for objects with more distinctive information. Within the same 200–300 ms time frame, Clarke et al. (2013) also observed that MEG signals increased for objects with weakly correlated features, reflecting increased processing for concepts whose semantic features are relatively weakly related, and so require increased integration demands. Thus, while the initial semantic effects reflected the processing of shared information, between 200 and 300 ms neural activity was sensitive to both the shared and distinctive aspects of a concept's meaning, whose integration enables coherent and specific conceptual representations.

Taken together, these two studies highlight that coarse semantic information about objects is rapidly processed within the first 150 ms, and is modulated by the degree of shared feature information associated with an object. As shared features (e.g. *has legs*) tend to be distributed across many different category or domain members, the rapidly formed representations in ventral temporal cortex may be detailed enough for distinguishing between different types or categories of object. Further, object-specific semantic information is processed after approximately 150–300 ms showing effects of both the shared and distinctive (together object-specific) semantic features and could drive within-category dissociations. Therefore, by using semantic feature-based models of object semantics and feature-based statistics, we can reveal the kinds of semantic information processed in the brain over time highlighting the necessity of testing cognitive measures derived from the stimuli against neural activity.

While these two MEG studies uncover what kinds of semantic information are processed across time, they do not tell us about the neural mechanisms – feedforward, feedback and recurrent – that underpin the formation of increasingly specific conceptual representations. We have addressed this question using MEG by contrasting neural responses between a task requiring the recognition of the specific object (basic-level naming) with one involving a shallow semantic judgement (domain naming, i.e. living vs. nonliving). Prior fMRI research points to the fact that when recognising objects at a relatively shallow level of semantic detail, such as deciding if the object is living or non-living, brain activation is restricted to the posterior parts of the ventral temporal cortex, while accessing more fine-grained semantic representations, e.g. knowing the picture is a tiger, activates both posterior and anterior medial aspects of the ventral stream (Moss, Rodd, Stamatakis, Bright, & Tyler, 2004; Tyler et al., 2004). This research shows that brain activity is modulated by the detail of semantic information required during recognition. However, critical issues remain – how is the time-course of activity in the ventral stream modulated by accessing semantic knowledge at different levels of specificity, and how does this modulate feedforward and recurrent interactions within the ventral stream?

**Figure 3.**
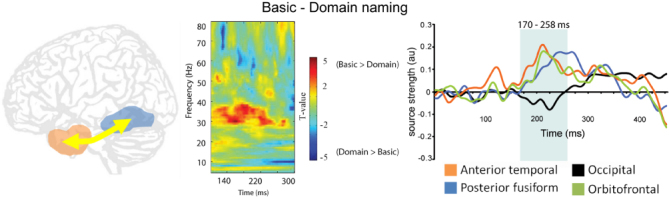
Recurrent interactions between the left anterior temporal and posterior fusiform increase when more specific semantic information is required. Left: Increased phase-locking between these regions during basic (e.g. tiger) compared to domain naming (i.e. living or nonliving). Right: increased activity in the anterior temporal lobe peaks ~200 ms and posterior fusiform peaks ~250 ms. Redrawn from Clarke, Taylor, and Tyler (2011).

Clarke, Taylor, and Tyler (2011) addressed these issues by recording MEG signals while participants recognised the same objects in two tasks requiring semantic knowledge at different levels of specificity. Theoretically, identifying an object as either a living or nonliving thing can be achieved based on information about shared semantic features alone, without the need to integrate the more distinctive properties into the emerging representation. Specific object identification requires exactly this – integrating the shared and distinguishing object properties, and so there is an increase in the semantic integration demands for recognition. Clarke et al. (2011) found no differences in neural activity during the first ~150 ms of basic and domain naming of objects, which may imply an equivalent early stage of processing of shared semantic information (required for both tasks) during a predominantly feedforward stage of visual object processing. After approximately 150 ms, increasing functional connectivity was apparent between the left anterior temporal and posterior fusiform regions when object-specific semantic information was required (Figure 3). The modulated functional connectivity during basic-level naming coincided with enhanced activity in the left anterior temporal lobe which was subsequently followed by increased activity in the more posterior fusiform. Therefore, the timing of the effects (post ~150 ms) and the anterior to posterior propagation of increased amplitude supports a hypothesis whereby recurrent processes are modulated by the relative need for more complex semantic feature integration. This suggests that when more specific conceptual properties are required, increased interactions between anterior and posterior temporal lobes act to bind together these semantic properties into coherent conceptual representations.

## The time-course of information processing during meaningful object recognition

Broadly, object representations undergo a transition in the first half-second, becoming increasingly fine-grained and specific (Clarke et al., 2013; Hegdé, 2008; Hochstein & Ahissar, 2002; Large, Kiss, & McMullen, 2004; Macé, Joubert, Nespoulous, Fabre-Thorpe, & Herzog, 2009; Martinovic, Gruber, Muller, & Lauwereyns, 2008; Schendan & Ganis, 2012; Sugase, Yamane, Ueno, & Kawano, 1999). As we saw above, this requires highly dynamic and interactive brain mechanisms through which visual information accumulates and cognitive operations are rapidly resolved across multiple time scales, with a continuing interplay between visual and cognitive factors (Humphreys & Forde, 2001; Humphreys, Riddoch, & Quinlan, 1988). Given the insights provided by the three previous examples, I will turn to addressing the principle issues raised in the beginning: how the initial perceptual information processing states become increasingly abstract and semantic over time, what is the nature of this semantic information and how do semantic representations change over time.

### Rapid perceptual to semantic effects

The earliest cortical signatures of visual processing are known to arise from V1. Within 100 ms of seeing an object, MEG and EEG studies using humans have shown that these initial responses are modulated by the low-level perceptual characteristics of the image (Clarke et al., 2013; Martinovic et al., 2008; Ramkumar, Jas, Pannasch, Hari, & Parkkonen, 2013; Scholte, Ghebreab, Waldorp, Smeulders, & Lamme, 2009; Tarkiainen, Cornelissen, & Salmelin, 2002), and are well modelled with computational models of V1 function (Clarke et al., 2014).

Within 150 ms, information has propagated anteriorly along the ventral temporal cortex (Bullier, 2001; Lamme & Roelfsema, 2000) where increasingly higher-level visual information is processed. Intracranial recordings in human ventral temporal cortex within this time frame show different response profiles for objects from different superordinate categories (Chan et al., 2011; Liu, Agam, Madsen, & Kreiman, 2009) that also display some degree of size and position invariance (Isik, Meyers, Leibo, & Poggio, 2014; Liu et al., 2009). These findings converge with EEG evidence showing categorical object distinctions are present within 150 ms (Schendan, Ganis & Kutas, 1998; Thorpe, Fize, & Marlot, 1996; VanRullen & Thorpe, 2001). Further, such rapidly processed categorical information can drive rapid behavioural responses (Crouzet, Kirchner, & Thorpe, 2010; Kirchner & Thorpe, 2006) presenting a challenge to our understanding of the neural mechanisms underpinning this cognitive process. While these studies show that higher-level visual information has been computed, more direct evidence is provided by Clarke et al. (2014) who, using MEG, revealed the processing of higher-level visual properties in humans prior to 150 ms and uncovered by the relationship between a computational model of higher-level visual processing and MEG signals.

Rapidly activated invariant visual responses are required to generalise away from the specific image being viewed. Such stimulus-abstracted visual processing may provide the foundation of semantic activation, as understanding the meaning of a visual image requires that stimulus independent information is processed. While the above evidence suggests that stimulus-abstracted object information can be accessed very rapidly, the nature of this object information, and whether semantic information is also rapidly accessed cannot be ascertained on this evidence.

Strong evidence that semantic information becomes available very rapidly comes from a word-picture interference study. Dell'Acqua et al. (2010) used EEG to compare neural signals from semantically related word–picture presentations with semantically unrelated word–picture presentations, with any observed effects of semantic relatedness depending on participants having accessed semantic information about both the word and the picture. They report rapid semantic effects, peaking at 106 ms, which suggests that stimulus-independent semantic information is accessed very rapidly, and is consistent with models of word production that claim conceptual knowledge is rapidly accessed (Levelt, Praamstra, Meyer, Helenius, & Salmelin, 1998). Such rapid semantic activation, in conjunction with higher-level visual responses, may underpin the reliable decoding of object category from MEG, EEG and intracranial recording studies (Cichy, Pantazis & Oliva, 2014; Chan et al., 2011; Liu et al., 2009; Murphy et al., 2011; Simanova, van Gerven, Oostenveld, & Hagoort, 2010) and dove-tails with evidence that categorical decoding from MEG signals is supported by semantic-feature models over and above that which can be accounted for by computational models of vision (Clarke et al., 2014).

### Nature of rapid semantic information

While these findings point to the rapid activation of object semantics, and provide evidence that semantic feature models are able to account for rapid neural activity, a more precise characterisation of rapid semantic responses has been provided through the use of semantic feature-based statistics. Clarke et al. (2013) related neural activity to particular aspects of semantic processing, derived from feature-based statistics, and found a relationship between early neural activity along the extent of the ventral processing stream and the degree of shared semantic information associated with objects. As shared features tend to be distributed across many different category or domain members, the rapidly formed representations in ventral temporal cortex may be detailed enough for distinguishing between different types or categories of object. Further, Hauk et al. (2007) reported that neural signals are rapidly modulated by an objects correlated feature structure. These studies show that rapid semantic effects seen in other studies may be underpinned by information processing of shared and correlated semantic object features – only uncovered using the predictions of feature-based cognitive models of semantic knowledge.

Further, we can speculate on the mechanisms by which this may occur. Higher-level visual information may activate partial semantic information that experience has associated with particular higher-level visual properties of the image. Further, additional semantic information will become activated if it frequently co-occurs with the initially activated information, resulting in a wealth of semantic information becoming active at the same time. As co-activated features tend to represent semantic information that regularly go together, and features that often co-occur tend to be found in many objects from a particular superordinate category (e.g. *has eyes*, *has ears*, *has legs* will co-occur together and occur in many animals), the initial semantic information that is activated will provide a bias towards concepts from a particular category but not the specific conceptual identity of the object. The use of computational models of semantics to simulate such processes, before testing the outputs of the model against the observed neural activity may provide a mechanism by which such predictions may be tested.

More broadly, the evidence discussed here allows us to claim that the initial transition from purely perceptual to coarse semantic processing begins very rapidly, and emerges as neural activity automatically propagates along the ventral temporal cortex. Further, this rapid activation of semantic information can underpin rapid categorical behaviours, but not concept-specific identification. The notion that rapidly activated object information supports coarse semantic representations that are built off the back of higher-level visual representations is also suggested in other models of the time-course of object recognition (Humphreys & Forde 2001; Schendan & Ganis, 2012). For example, the hierarchical interactive theory (Humphreys & Forde, 2001) claims that following the initial visual processing of an object, there is a cascade-like sequence of processing where the initial perceptual processing rapidly activates (some) semantic information associated with the object. The rapidly activated semantic information (including non-visual information) then continues to interact with the ongoing perceptual processes. What we have described above adds additional details such as the timings of rapid semantic effects while crucially also uncovering the type of semantic information processed.

### How do semantic representations change over time: object-specific semantic effects and the anterior medial temporal lobe

After ~150 ms, conceptual object representations become increasingly specific and fine-grained, with this temporal progression of conceptual specificity also observed in behavioural studies (Fei-Fei, Iyer, Koch, & Perona, 2007; Mace et al., 2009; Mack, Gauthier, Sadr, & Palmeri, 2008). Using MEG we have shown that neural activity increased after 150 ms when participants named objects at a specific level compared to a general category level (Clarke et al., 2011), and that specific objects from the same superordiante category can be successfully distinguished by a model of the MEG signals based on semantic features (Clarke et al., 2014). These findings are in line with other studies showing that concept-level information is represented and processed beyond 150–200 ms (Low et al., 2003; Martinovic et al., 2008; Schendan & Maher, 2009). The timing of these object-specific effects suggests that the formation of detailed semantic representations is not accomplished within the timeframe of the initial propagation of signals along the ventral temporal cortex, but is dependent on more dynamic recurrent processing mechanisms.

A key region implicated in the formation of specific-conceptual representations is the perirhinal cortex within the anterior medial temporal lobes (Clarke & Tyler, 2014; Moss et al., 2004; Tyler et al., 2013, 2004). The perirhinal cortex is claimed to code for complex conjunctions of simpler information in posterior ventral temporal regions (Barense et al., 2012; Buckley, Booth, Rolls, & Gaffan, 2001; Bussey & Saksida, 2002; Murray & Richmond, 2001), and may code the computations necessary for object-specific semantic representations to be formed (also see Damasio, 1989; Meyer & Damasio, 2009; Rogers & Patterson, 2007; Simmons & Barsalou, 2003). This hypothesis is supported by human fMRI research showing that activity in the region is sensitive to both object-specific semantic content (Clarke & Tyler, 2014) and feature-based statistics capturing semantic feature integration (Tyler et al., 2013), in addition to the integration of complex semantic information (Barense, Rogers, Bussey, Saksida, & Graham, 2010; Kivisaari, Tyler, Monsch, & Taylor, 2012; Moss et al., 2004; Tyler et al., 2004; Taylor, Moss, Stamatakis, & Tyler, 2006; Taylor, Stamatakis, & Tyler, 2009).

Using MEG, it has been observed that the anterior temporal lobes^1^ are sensitive to object-specific semantics beyond 150 ms, along with anterior and posterior interactions within the temporal lobe (Campo et al., 2013; Clarke et al., 2011; Clarke et al., 2014; Urooj et al., 2014) that may underpin the formation of specific semantic representations (requiring the integration of distinctive feature information into the established categorical context). Widespread damage to the anterior temporal lobe is associated with impairments in accessing specific semantic knowledge (Mion et al., 2010; Rogers & Patterson, 2007). Further, atrophy in the anterior temporal lobes is associated with reduced activation in the posterior ventral temporal cortex during semantic decisions (Mummery et al., 1999), while lesions to the rhinal cortex (perirhinal and entorhinal) and temporal pole results in reduced backwards connectivity from the anterior temporal lobe to the posterior ventral stream during object recognition (Campo et al., 2013). Overall, these studies strongly support the fundamental role of the perirhinal cortex, within the anterior temporal lobes, in the formation of object-specific semantic representations through processing conjunctions of coarser information represented in the posterior fusiform. Further, such object-specific semantic information is integrated through recurrent connectivity between posterior and anterior sites in the ventral stream with such processes beginning after approximately 150–200 ms.

In terms of activating concept-specific semantic information, we can speculate that after the initial phase where many semantic features become activated (providing a strong category bias but not clear information for object-specific representations), the perirhinal cortex's role is to bind and integrate the conceptual features that will form a coherent and specific conceptual representation. Integrating semantic features, and especially the most weakly correlated distinctive features, is critical for disambiguating between otherwise similar conceptual representations (Randall et al., 2004; Tyler et al., 2013). Further, MEG evidence of the conjoint processing of shared and distinctive features along with weakly correlated features between 200 and 300 ms (Clarke et al., 2013) shows that information processing during this time frame is sensitive to the conceptual properties of objects that are required for specific representations to be formed.

## Discussion

The above account is concerned with explaining the kinds of cognitive and functionally relevant information that is processed when recognising meaningful visual objects. In particular, it is primarily concerned with how the initial sensory signals undergo a series of information processing states to establish a specific conceptual representation. One key finding is that semantic information about objects is rapidly activated, and that early signatures of semantic information can drive coarse, superordinate categorical, judgements about objects. Further, rapid semantic processes (and behaviours) are underpinned by shared feature information. Post ~150 ms, a more dynamic, interactive, phase of processing begins that underpins the formation of more specific concept-level representations. This is dependent on the integration of semantic information, particularly the integration of distinctive information into the prior categorical context, and is underpinned by interactions between the anterior temporal lobe, specifically the perirhinal cortex, and the posterior fusiform.

Our research shows clear evidence that semantic information plays a key role during the temporal formation of object representations. Further, we suggest that unique conceptual representations are not established within an initial feedforward sweep of processing along the temporal lobes but depend on recurrent interactions within the ventral stream. Such a progression of semantic information processing over time need not imply discrete stages of initial category representations and subsequent object-specific semantic representations, but can be formulated in terms of different kinds of information emerging and accumulating over time (Mack & Palmeri, 2011).

Previous MEG and EEG research has been able to indentify time frames, neural regions and oscillatory dynamics associated with semantic processing of objects. The multistate interactive (MUSI) account of object cognition (Schendan & Ganis, 2012; Schendan & Maher, 2009; Schendan & Stern, 2008) proposes that perceptual categorisation occurs between 100 and 150 ms and precede concept-level semantic processing that is driven by recurrent interactions between frontal, temporal and parietal regions. While the current account shares many aspects with the MUSI account, the critical advance we make, by combining quantifiable accounts of object semantics with MEG signals, is to explicitly relate different types of semantic information to time-varying neural signals and track the progression of semantic information processing over time. Uncovering such information processing states would not be possible without a detailed model of object semantics that incorporates information about an objects semantic content and the statistical regularities in the co-activation of semantic information.

The account described here shows that detailed semantic representations can be formed within 300 ms, and that are sufficiently specific to support the unique identification of the object. The N400, an electrophysiological signature beginning around 200/300 ms and peaking after 400 ms, is widely seen as a marker of the integration and access of semantic memory (for review see Kutas & Federmeier, 2011). Here, we reviewed evidence for concept-specific semantic integration with a similar onset to that of N400 effects, but crucially our data show that specific forms of semantic information are rapidly accessed prior to effects within the N400 time range. Here we have shown that there are important rapid semantic effects that can in turn constrain models of subsequent effects that may include N400-like processes, the resolution of lexical and phonological processes (Indefrey & Levelt, 2004; Levelt et al., 1998), and word selection and production (Riès, Janssen, Burle, & Alario, 2013). This is not to claim that these cognitive operations can only begin after the completion of conceptual processes, but likely begin prior to completion of preceding phases as suggested by cascaded models of recognition (Hauk, Davis, Ford, Pulvermuller, & Marslen-Wilson, 2006; Humphreys & Forde, 2001). Understanding the first few hundred milliseconds of information processing provides a crucial platform for understanding subsequent effects which can only be fully understood once there is an account of the preceding phases of processing.

While beginning to unravel the information processing states associated with transitions and early interplays between perception and semantics, many important aspects of meaningful object recognition remain unclear – such as the role of network connectivity in information transitions and the functional role of different oscillatory frequencies. Further, what are the contributions of a wider network of regions engaged during object recognition – including interactions with the frontal lobe. Bar and colleagues (Bar et al., 2006; Ghuman, Bar, Dobbins, & Schnyer, 2008) have shown early frontal – temporal interactions during object recognition and processing contextual associations, which may form parallel interactive processing streams together with those described here in the service of semantic memory (also see Schendan & Stern, 2008).

In conclusion, the extraction of meaningful information from visual objects relies on a dynamic sequence of neural activity and regional inter-activity. The semantic representation of an object emerges from a relatively coarse state, supporting broad discriminations between different types of things, to a fine-grained semantically rich integrated representation. This evolution of meaning from a perceptual to conceptual form relies on feedforward and recurrent processing mechanisms, along with the dynamic interactions between brain regions supporting object recognition.

## Note

1. While human fMRI studies indicate the perirhinal cortex underlies object-specific conceptual processing, MEG does not provide the spatial resolution to accurately make inferences about the specific anatomical structure within the anterior temporal lobes the effects originate from. Therefore, we interpret our MEG effects at the level of the anterior temporal lobe and rely on techniques with increased spatial specificity to more accurately constrain our regional hypotheses.
